# CHEMICAL LEUCODERMA SYMPOSIUM

**DOI:** 10.4103/0019-5154.70673

**Published:** 2010

**Authors:** A K Bajaj

**Affiliations:** *From the Department of Skin, V.D and Leprosy, M.L.N. Medical College, Allahabad, Uttar Pradesh, India*

Leucoderma due to contact with chemicals was reported for the first time in 1939 in factory workers using acid-cured gloves. Mono-benzyl ether of hydroquinone (MBH) was implicated as the causative agent. There were no reports of chemical leucoderma in the English literature for the next three decades though some reports did appear in the Russion and Japanese literature in the 1960s. In the 1970s–1980s, many landmark studies were carried out implicating various phenols and catechols as the causative agents. Due to stringent controls in the factories, the incidence of chemical leucoderma came down considerably. In the early 1990s, commonly used chemicals such as paraphenylene diamine (PPD) was documented to cause depigmentation. In India, chemical leucoderma is common. Most of the reported cases are due to consumer products such as bindis (*para*-tertiary butyl phenol), footwear (MBH in rain shoes and now fancy plastic chappals), synthetic wallets (MBH), hair dyes (PPD), alta (azo dyes), etc. Due to poor quality control, the incidence of chemical leucoderma due to consumer items has gone up. The classical example is black henna (kali mehndi) used for dyeing the hair. Majority of the so-called herbal black hennas contain over 10% PPD which is a strong sensitizer as well as a depigmenting agent. Surprisingly, there are hardly any Indian reports of chemical leucoderma as an occupational hazard which could be due to lack of interest of dermatologists and ignorance as well.

This symposium has write-ups by authorities in the field, Dr. Sanjay Ghosh, Dr. A K Bajaj, and Dr. Abir Saraswat.

